# *Campylobacter* infection of broiler chickens in a free-range environment

**DOI:** 10.1111/j.1462-2920.2008.01623.x

**Published:** 2008-08

**Authors:** Frances M Colles, Tracey A Jones, Noel D McCarthy, Samuel K Sheppard, Alison J Cody, Kate E Dingle, Marian S Dawkins, Martin C J Maiden

**Affiliations:** 1 The Peter Medawar Building for Pathogen Research, Department of Zoology, University of OxfordSouth Parks Road, Oxford OX1 3SY, UK; 2 Department of Zoology, University of OxfordSouth Parks Road, Oxford OX1 3PS, UK; 3 Nuffield Department of Clinical Laboratory Sciences, Oxford University, John Radcliffe HospitalOxford OX3 9DU, UK; 4 Department of Microbiology, Oxford Radcliffe NHS Trust, Level 6 Microbiology, John Radcliffe HospitalOxford OX3 9DU, UK

## Abstract

*Campylobacter jejuni* is the most common cause of bacterial gastroenteritis worldwide, with contaminated chicken meat considered to represent a major source of human infection. Biosecurity measures can reduce *C. jejuni* shedding rates of housed chickens, but the increasing popularity of free-range and organic meat raises the question of whether the welfare benefits of extensive production are compatible with food safety. The widespread assumption that the free-range environment contaminates extensively reared chickens has not been rigorously tested. A year-long survey of 64 free-range broiler flocks reared on two sites in Oxfordshire, UK, combining high-resolution genotyping with behavioural and environmental observations revealed: (i) no evidence of colonization of succeeding flocks by the *C. jejuni* genotypes shed by preceding flocks, (ii) a high degree of similarity between *C. jejuni* genotypes from both farm sites, (iii) no association of ranging behaviour with likelihood of *Campylobacter* shedding, and (iv) higher genetic differentiation between *C. jejuni* populations from chickens and wild birds on the same farm than between the chicken samples, human disease isolates from the same region and national samples of *C. jejuni* from chicken meat.

## Introduction

*Campylobacter jejuni* is considered to be the most common cause of bacterial gastroenteritis globally, with an annual estimated disease burden of 2.5 million cases in the USA and in excess of 340 000 cases in the UK (Allos, 2001; Kessel *et al*., 2001). In industrialized countries, the majority of cases are self-limiting relatively mild cases of gastroenteritis, although the economic impact of the large number of these is substantial (Ketley, 1997; Withington and Chambers, 1997; Roberts *et al*., 2003). Although there has been some success in understanding and controlling disease caused by pathogens such as *Salmonella enterica* phage type 4 and *Escherichia coli* O157:H7, other pathogens, especially *C. jejuni*, continue to represent major, yet poorly understood, health risks. The sporadic nature of most cases of campylobacteriosis and the ubiquitous presence of *C. jejuni* and its close relative *Campylobacter coli* in the gastrointestinal tract and faeces of many animal and bird species, combined with difficulties in the typing of isolates, have hampered the identification of infection sources (Dingle *et al*., 2001; Frost, 2001; Newell and Fearnley, 2003; Rivoal *et al*., 2005). Consequently the relative contributions of various potential sources of human infection have yet to be established definitively.

The frequent contamination of retail chicken meat with high numbers of *C. jejuni* implicates this as a major source of human infection (Harrison *et al*., 2001; Moore *et al*., 2002). Consequently, health authorities have placed a major emphasis on reducing the contamination of such products. Some success has been achieved in reducing the extent of *C. jejuni* shedding by chickens reared in intensive, housed, production facilities by the implementation of biosecurity measures (Humphrey *et al*., 1993). However, these are rarely completely effective and, at a time when free-range and organically raised birds are becoming more popular with consumers for welfare reasons, there is currently no strategy for the reduction of the burden of infection in extensively produced chickens, which normally shed campylobacters in their faeces at high rates (Heuer *et al*., 2001; Rivoal *et al*., 2005).

Colonization of food animals, specifically free-range chickens, with *Campylobacter* from their environment is widely thought to be a likely route of infection (Newell and Fearnley, 2003; Bouwknegt *et al*., 2004; Rivoal *et al*., 2005; Bull *et al*., 2006; Huneau-Salaun *et al*., 2007). However, the high diversity and wide distribution of *C. jejuni* populations in wild and farm animals has seriously complicated the investigation of infection sources. Even where high-resolution nucleotide sequence-based typing is appropriately applied, establishing sources of infection is inherently difficult as the failure to detect the presence of a particular genotype in the immediate vicinity of a production facility does not prove that the organism is not present at levels too low to be detected microbiologically while sufficiently high to act as an infection source. Conversely, the presence of a genotype in the environment does not in itself prove a source of contamination or route of transmission.

To address this problem, we tested three independent hypotheses arising from the assumption that environmental contamination is responsible for the high levels of *Campylobacter* shedding observed in free-range chickens, namely: (i) that the *Campylobacter* genotypes isolated from free-range chicken crops will be clustered, with succeeding flocks on the same range contaminated with the same genotypes of *Campylobacter* observed in preceding flocks, (ii) that the extent of colonization of the free-range chickens will be influenced by their ranging behaviour, and (iii) that wild birds in the vicinity of the range will be colonized with *Campylobacter* populations related to those isolated from the free-range chickens. A survey of the shedding of *Campylobacter* was undertaken in 64 flocks of commercial free-range broiler chickens which were part of an ongoing study in which their health, performance and ranging behaviour were studied in relation to experimental variation in tree cover and stocking density (Jones *et al*., 2007). The results provided no evidence that the free-range environment represents a major source of contamination, indicating that other potential sources of chicken colonization should be more carefully examined.

## Results

### Dynamics of *Campylobacter* infection in free-range chicken flocks

Flocks 1 and 57 were sampled for *Campylobacter* at weekly intervals from 7 to 56 days of age. *Campylobacter* was first detected in flock 1 on day 35, when all 10 birds sampled were positive, and all birds tested were positive until depletion at day 56. *Campylobacter* was first detected in flock 57 on day 28 when 91 (91%) birds tested were positive, and between 80% and 100% birds tested were positive each week until depletion. In both flocks the positive status coincided with the birds being let onto the range for the first time after being moved from brooding arcs to rearing arcs.

A total of 549 *Campylobacter* isolates were characterized by multi-locus sequence typing (MLST), giving a total of nine sequence types (STs), four from flock 1 and five from flock 57, with no sharing of genotypes between the two flocks. In both flocks the number of STs isolated was greater at day 56 than when *Campylobacter* was first detected, when one or two genotypes dominated. Both flocks were colonized by multiple genotypes, with up to three being isolated in a single time point from crop 1 and up to five being isolated in a single time point from flock 57.

### Survey of *Campylobacter* infection of 64 free-range chicken flocks

A total of 975 chickens from 64 flocks were sampled prior to depletion over a period of a 10 months (February to December 2003), yielding 881 *Campylobacter* isolates (shedding rate 90.4%) of which 420 (47.7%) were *C. jejuni*, 448 (50.9%) were *C. coli* and 13 (1.3%) unspeciated (Table 1). With the exception of 13 isolates that could not be isolated as pure cultures, all isolates were characterized by MLST, giving a total of 31 different STs with a Simpson's index of diversity of 0.91 (Hunter, 1990). The STs clustered into 10 groups of related genotypes, known as clonal complexes, eight of which were characteristic of *C. jejuni* and two of *C. coli*. The most common genotypic group was the *C. coli* ST-828 complex (39.9% of isolates), followed by the *C. jejuni* ST-661 complex (14.3% of isolates). Shedding rates and diversity were similar between two farms.

**Table 1:** *Campylobacter* shedding from 64 successive flocks sampled at Wytham and Northmoor.

				Clonal complex (frequency)											
Flock	Plot	Date	No. samples positive/total	21	48	45	257	460	573	574	661	828	1150	UA	UT
1	1	10.02.2003	100/100	20	2							78			
2	2	14.02.2003	9/10	4								5			
3	3	21.02.2003	9/10									9			
4	4	21.02.2003	5/10	3								2			
5	5	24.02.2003	8/10	8											
6	6	07.03.2003	10/10	10											
7	7	14.03.2003	5/10	5								5			
8	8	14.03.2003	8/10	6								1			1
9	9	21.03.2003	10/10	1	1	5						3			
10	10	28.03.2003	9/10		1						3	5			
11	11	04.04.2003	9/10	6	3										
12	12	04.04.2003	9/10		1						6	2			
13	13	11.04.2003	10/10	1	3				1			5			
14	14	17.04.2003	10/10					1	3		5			1	
15	15	25.04.2003	10/10		1							9			
16	16	25.04.2003	9/10						1		2	7			
17	1	02.05.2003	10/10	1	1						5	3			
18	2	09.05.2003	10/10	2						2		6			
19	3	16.05.2003	10/10	7								3			
20	4	16.05.2003	10/10	6								4			
21	5	23.05.2003	10/10		4							6			
22	6	30.05.2003	10/10	2						2		6			
23	7	06.06.2003	10/10	1						1		8			
24	8	06.06.2003	7/10	2	1					1		2		1	
25	9	13.06.2003	10/10	1						2		6	1		
26	10	20.06.2003	9/10		1		4					4			
27	11	27.06.2003	6/10				3					3			
29	13	03.07.2003	5/9		1							4			
30	14	11.07.2003	10/10		2				2	1		5			
31	15	18.07.2003	10/10				4					6			
32	16	18.07.2003	10/10									10			
33	1	25.07.2003	10/10				1		2			5	1		1
34	2	01.08.2003	7/10	1					1			3	1		1
35	3	08.08.2003	8/10								1	5	2		
36	4	08.08.2003	8/10	1								5	2		
37	5	15.08.2003	8/10		1						1	5			
38	6	22.08.2003	6/10							2	1	2	1		
39	7	29.08.2003	9/10								3	3		3	
40	8	29.08.2003	9/10								2	2	1	4	
41	9	05.09.2003	10/10									8		2	
42	10	12.09.2003	10/10						3			6			1
43	11	19.09.2003	10/10						1		2	6	1		
44	12	19.09.2003	10/10								1	6	3		
45	13	26.09.2003	7/10								3	4			
46	14	03.10.2003	8/10						1		3	2	1		1
47	15	10.10.2003	10/10								1	9			
48	16	10.10.2003	9/10						2	1	3	3			
49	1	17.10.2003	9/10						7			2			
50	2	24.10.2003	10/10						1		1			8	
51	3	31.10.2003	8/10	1					7						
52	4	31.10.2003	10/10	1					3		6				
53	5	07.11.2003	18/25	4							2	12			
54	6	14.11.2003	23/25	4					1		2	15			1
55	7	21.11.2003	22/25	1					16		1	3			1
56	8	21.11.2003	20/23	7					1		3	9			
57	9	28.11.2003	91/100	6					32	15	6			32	
58	10	05.12.2003	18/25						4			7		7	
59	11	12.12.2003	23/25							1	2	1		19	
60	12	12.12.2003	23/25							1	4	3		13	2
61	13	19.12.2003	25/25	3					7		8	5			2
62	14	19.12.2003	21/25						11	1	6	3			
63	15	19.12.2003	24/25								17	5			2
64	16	19.12.2003	25/25							1	24				

UA, STs that could not be assigned to a clonal complex; UT, isolates that were not typed by MLST.

In addition the *flaA* SVR was sequenced to give added resolution. A total of 29 *flaA* SVR types were identified among the 868 *Campylobacter* isolates, giving 64 different combinations of ST-*flaA* SVR. The *flaA* SVR type was loosely associated with ST but could not be entirely predicted. Nineteen of the 31 STs (60%) were associated with only one *flaA* SVR type, but the remainder were found in combination with up to eight *flaA* SVR types of varying peptide as well as nucleotide type. Conversely some *flaA* SVR types were found in association with more than one ST or clonal complex.

### Clustering of *Campylobacter* genotypes

For free-range chickens the same ST-*flaA* SVR types were isolated on days that averaged 61 days apart. For different ST-*flaA* SVR types the average was 104 days (*P* < 0.0001). For starlings the times were 93 and 144 days respectively (*P* = 0.05).

Over the course of the survey, 16 plots were used at the two farm sites, with four successive flocks raised on each plot. There were 39 occasions on which an identical genotype was identified on the same plot. Under the null hypothesis of no carry-over of genotypes, a permutation test showed that the expected number of identical genotypes by chance alone was 38, with a standard deviation of 3. The genotype present in a given flock could not be therefore predicted from the genotype shed by the previous flock on the same plot, despite the inevitable contamination of the plots with chicken faeces.

### Impact of behaviour on *Campylobacter* shedding

**Fig. 1:**
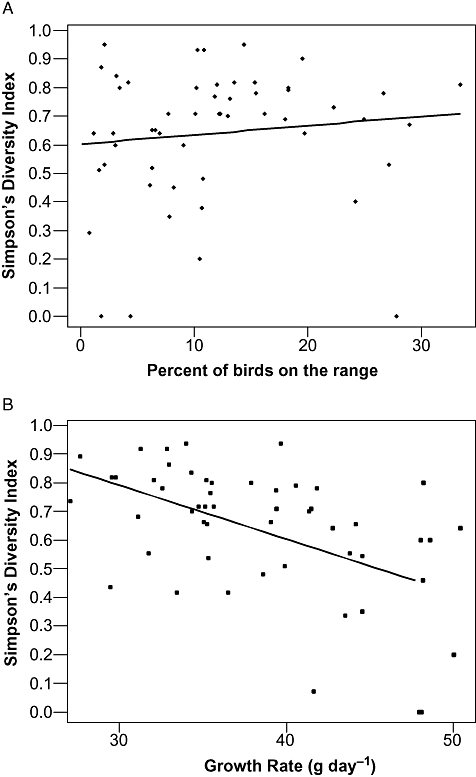
Correlation of *Campylobacter* diversity with (A) percentage of birds ranging and (B) growth rate and hock burn. There was no correlation between *Campylobacter* diversity and ranging behaviour. Increased diversity of *Campylobacter* genotypes was positively correlated with decreasing growth rate and improving hock health. A. Diversity = 0.602 + 0.003% birds on range. S = 0.233, R-Sq = 1.3%, R-Sq (adj) = 0.0%. B. Diversity = 1.374 − 0.019 growth rate. S = 0.207, R-Sq = 23.9%, R-Sq (adj) = 22.3%.

*Campylobacter* shedding rates and diversity were not affected by chicken density or tree presence, neither were they correlated to chicken ranging behaviour, or flock mortality, gait and foot pad dermatitis (Fig. 1A). *Campylobacter* diversity however was significantly affected by average flock growth rate (*F*_7,51_ = 14.0, *P* = 0.001, *r* = −0.49) and incidence of worse hock burn (score 2, *F*_7,51_ = 7.4, *P* < 0.01, *r* = −0.39), so that diversity increased as growth rate and worse hocks decreased (*R*^2^ = 39.7%) (Fig. 1B). Therefore, while there was no evidence of ranging behaviour affecting *Campylobacter* shedding or diversity, there was some evidence of chicken health being correlated with *Campylobacter* diversity.

### *Campylobacter* isolated from wild birds

*Campylobacter* were isolated and characterized from fresh faeces collected from wild geese species and starlings (*Sturnus vulgaris*) in the locality of the Wytham plots during the survey. A total of 166 *C. jejuni* isolates were obtained from 331 samples of geese faeces, corresponding to a shedding rate of 50.5%: only one *C. coli* was isolated from wild geese. From these isolates 38 STs were identified, which clustered into five clonal complexes, the most common being ST-1034 complex (15.7% of isolates) and ST-702 complex (12.0% of isolates). A total of 75 STs were identified from 293 *C. jejuni* isolates from 964 starling samples (shedding rate, 30.4%): only six *C. coli* and 60 *C. lari* were isolated from wild starlings. The *C. jejuni* isolates clustered into 11 clonal complexes, of which ST-682 complex (50.0% of isolates) and ST-177 complex (24.8% of isolates) were the most common.

Additional sequencing of the *flaA* SVR gave 23 alleles and 47 ST-*flaA* SVR genotypes among the isolates from geese and 54 alleles and 125 ST-*flaA* SVR genotypes among the isolates from starlings. Three *flaA* SVR alleles were isolated in low frequency from geese and starlings, but no isolates identical by ST and *flaA* SVR were isolated from both wild bird species.

### Comparison of ST-*flaA* SVR fine types

**Fig. 2:**
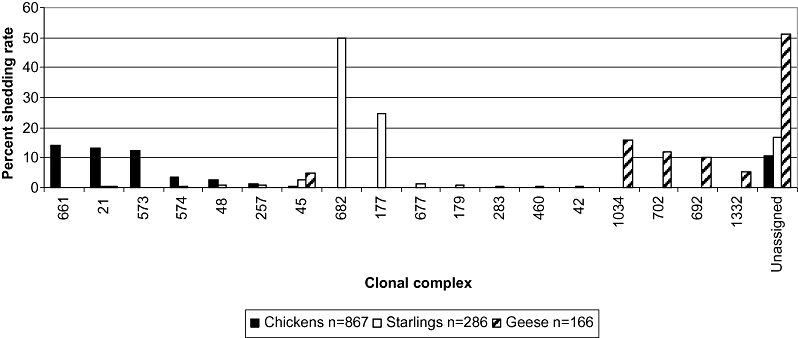
Abundance curve of *C. jejuni* clonal complexes isolated from wild birds and free-range chickens.

Five of the 19 clonal complexes identified among the two wild bird species and the domestic chickens overlapped (Fig. 2). Four STs (ST-45, ST-257, ST-574 and ST-1023) were isolated from both the chickens and starlings, but no STs isolated from geese were identified among chickens or starlings. Two *Campylobacter* isolates with identical ST and *flaA* SVR (ST-257; 16-12 and ST-1023; 80-48) were isolated in low frequency from chickens and starlings, but time of isolation was not contemporary, with the strains isolated 6–18 months later among starlings.

Eight of 10 *Campylobacter* clonal complexes isolated from the free-range chickens were among the nine complexes most commonly isolated from chicken meat sampled in the UK, accounting for 76.5% of meat isolates. The sixth most common complex accounting for 5.3% of meat isolates, ST-443 complex, was not isolated from the free-range chickens and the *C. coli* ST-1150 complex isolated from free-range chickens was absent from the chicken meat samples. Of the 31 STs isolated from the free-range chickens, 19 (61.3%) were isolated from the chicken meat samples. Nearly a third (*n* = 20) of the 61 *Campylobacter* ST-*flaA* SVR strains isolated from the free-range chickens were identified among the chicken meat isolates, accounting for 204 of 1003 (20.3%) isolates.

None of the *C. jejuni* isolates from geese had the same ST-*flaA* SVR type as those isolated from human disease. Five of the STs identified among isolates from starlings had the same ST-*flaA* SVR type (3.5% of isolates) as those isolated from human disease (11.6% of isolates). Nine of the STs identified among isolates from free-range chickens (70.5% of isolates) had the same *flaA* SVR type as those isolated from human disease (18.4% of isolates).

### Genetic differentiation of *Campylobacter* isolates

Fisher's statistic (*F*_ST_) was used to compare the genetic similarity of the *C. jejuni* populations sampled with data for isolates obtained from human disease in Oxfordshire in 2003/2004 and from retail chicken meat in the UK in 2001 and 2005/2006 (Table 2). The *F*_ST_ value comparing the *Campylobacter* populations consisting of both *C. jejuni* and *C. coli* between the free-range chickens and chicken meat was 0.115, *P*-value < 0.001 and between the free-range chickens and Oxfordshire human disease was 0.143, *P*-value 0 < 0.001. Fisher's statistic was also used to compare the *Campylobacter* populations isolated from the free-range chickens at the two different farm sites, Wytham and Northmoor, giving a value of 0.036, *P*-value < 0.001. Using STRUCTURE analysis of all isolates, host species association of STs could be predicted with 97% accuracy. Using each ST only once for each species, the prediction accuracy dropped to 80% accuracy.

**Table 2:** Measure of gene flow (*F*_ST_) among *C. jejuni* isolates.

Host source	Chickens	Geese	Starlings	Chicken meat
Geese	0.204	–	–	–
Starlings	0.596	0.604	–	–
Chicken meat	0.148	0.251	0.408	–
Human disease	0.101	0.276	0.467	0.034

Free-range broiler chickens, starlings, geese and human disease cases were sampled in Oxfordshire; chicken meat was sampled nationally.

## Discussion

Reducing the contamination of food by pathogens plays a central role in improving and maintaining public health. In achieving this, an understanding of pathogen distribution and transmission is required to drive policy development and implementation, and to direct consumer choice. In the case of human campylobacteriosis, however, gaining this fundamental understanding has been hindered by the genetic and antigenic diversity of *C. jejuni* and *C. coli*, combined with their ubiquitous distribution in farm and wild animals (Dingle *et al*., 2001; Frost, 2001). In the present investigation, a multidisciplinary approach was adopted. This examined various aspects of genotypic diversity in *Campylobacter* isolates obtained from a well-studied broiler chicken production facility over the period of a year. These data were used to test three hypotheses following from the assumption that free-range chickens are colonized from their environment. None of the data collected were consistent with the predictions arising from this assumption.

Exhaustive sampling of the environment is technically challenging as survival of *Campylobacter* outside of the host is limited and levels of contamination below that which can be detected in the laboratory cannot be excluded. In contrast, chicken flocks can be considered to be a highly sensitive indicator of environmental contamination. Notwithstanding the fact that the rearing arks were not microbiologically secure, in the two flocks when weekly samples were taken, the chickens did not commence shedding *Campylobacter* until they went onto the range at days 35 and 28, respectively, which would be expected if the range were a major source of infection. This further implied that the preceding flocks on the same range were a likely source of contamination of succeeding flocks as, although the rearing arks that house the chickens were cleaned between each flock and moved around the plots, no decontamination of the entire range was undertaken other than natural exposure to sunlight. There was, however, no evidence of carry-over of *Campylobacter* genotypes between flocks occupying the same plot and range, as predicted if contamination of the plot and range by preceding flocks was a source of *Campylobacter* infection. Further, the initial flocks were reared on land which had never been used to rear chickens previously, but they were still readily colonized. Housed chicken flocks also typically commence shedding of *Campylobacter* at this age and it may be that factors other than environmental contamination, such as changes in gut flora, immunity and hormones produced in response to stress, may influence the onset of shedding (Cogan *et al*., 2007; Humphrey, 2006).

Similar genotypes of *Campylobacter* were temporally clustered and this effect was substantially higher than can be explained by chance. While this could be interpreted as being consistent with environmental contamination, the effect was much less marked for wild starlings, although the observed results were less well supported statistically. Combined with the observation that identical genotypes were isolated from successive flocks reared on different plots and farm sites, this was consistent with successive flocks acquiring infection from common sources over a period of time. Other studies have observed farm-specific, as well nationally distributed, clones of *Campylobacter* and this may reflect contamination consequent from movement of animals, equipment or personnel, which could operate on a local as well as a national scale (Petersen and Wedderkopp, 2001; Karenlampi *et al*., 2003; Wittwer *et al*., 2005).

Chicken behaviours, such as ranging, may be expected to influence the extent of colonization by *Campylobacter* if the source was the free-range environment with, for example, a high degree of ranging by a flock exposing it to more sources of contamination and thus higher rates of colonization and a larger diversity of genotypes. Similarly, health aspects, such as incidence of foot pad dermatitis, may be interlinked by limiting ranging behaviour or promoting other behaviours such as coprophagy if mobility is restricted (ACMSF, 2004). There was no evidence of ranging behaviour or health affecting *Campylobacter* shedding rates, rather, the diversity of *Campylobacter* genotypes was significantly correlated with growth rate and less so with severity of hock burn, with diversity increasing with decreasing growth rate and improving hock condition. This is the first demonstration that specifically diversity, rather than shedding rate of *Campylobacter* among the flock, may be linked with chicken health.

Wild birds are thought to be an important source of infection if they gain access to housed birds, or by contaminating surface water or soil that is readily accessible to free-range birds (Newell and Fearnley, 2003; Sopwith *et al*., 2003). Migratory species could potentially spread *Campylobacter* strains over very large distances (Waldenstrom *et al*., 2002; Karenlampi *et al*., 2007). As such, wild birds and free-range poultry could be expected to have many *Campylobacter* genotypes in common. There was no evidence for these ideas from the two wild bird populations studied. The *Campylobacter* populations present in geese and starlings differed significantly from those isolated from the chickens, despite their close proximity, open access to the range and abundance on the farm. Just over 50% of isolates from the free-range chickens were *C. coli*, while the species was rare among both geese (0.6% of isolates) and starlings (1.7% of isolates). For the two populations studied, there was no support for the contention that wild birds are a major source of contamination of free-range chickens. Few clonal complex-containing clusters of related genotypes, and even fewer STs, were shared between the poultry and wild birds which was consistent with results from other studies using macrorestriction profiling (Petersen *et al*., 2001; Broman *et al*., 2002). Two *Campylobacter* isolates with identical ST and *flaA* SVR were isolated from the free-range chickens and starlings but they occurred at low frequency and were separated by a period of 6–18 months. There were no STs in common between the chickens and geese, and indeed between starlings and geese over the combined 3-year period, implying that each bird species has host-specific populations of *Campylobacter*, supporting results from a large study of migrating birds (Waldenstrom *et al*., 2002).

Isolates from the free-range poultry were compared with those from a national survey of retail chicken meat products (United Kingdom Food Standards Agency, Research Project B15011). There was less genetic differentiation between the free-range chickens and meat than between the chickens and the wild birds, implying that host source is more closely linked than temporal and geographical proximity. The greater effect of host over time or space was similarly demonstrated in a study comparing isolates from Dutch and UK chickens, where, for example, UK chickens had more similar *C. jejuni* to Dutch chickens than UK cattle, and this was upheld when comparing ‘early’ (1990–1997) and ‘late’ (1998–2003) isolates (McCarthy *et al*., 2007). It is notable that this occurs despite the influence of slaughter and processing plants, and the local production of the free-range chickens compared with the national distribution of retail chicken meat. Although not matched in time, eight of the nine clonal complexes, accounting for 76.5% of chicken meat isolates, were isolated from the free-range chickens, with almost a third of *Campylobacter* isolates being identical by both ST and *flaA* SVR type. The absence of ST-443 complex, common among chicken meat, from the free-range chickens may be a sampling issue; for example, it could be associated with housed rather than extensively reared birds. With sampling of live chickens on a larger scale it is likely that the overlap with chicken meat would be even greater. Unlike the wild bird samples, *C. coli*, and particularly those genotypes clustering into ST-828 complex, was common among the chicken meat samples, accounting for just over a quarter of them.

While there were statistically significant differences in the *F*_ST_ values for all of the comparisons, which were consistent with the differing sources and sampling regimes represented, there were differences in the magnitude of the *F*_ST_ values that were informative. The differences in *F*_ST_ for the comparison of the chicken meat and human disease *C. jejuni* isolates were relatively small (*F*_ST_ = 0.034 or 3.4%), which was consistent with retail chicken meat being a major, but not the only source, of human disease. The *F*_ST_ values for the comparisons of genotypes obtained from wild birds and broiler chickens were much larger (0.204–0.604), which indicated that each bird species had distinct *C. jejuni* populations. The *F*_ST_ value for comparisons of chickens at Wytham and Northmoor was low (0.036), providing further support that these shared a common source of infection, notwithstanding their geographical separation. The fact that broiler chicken *C. jejuni* population had the lowest *F*_ST_ values in comparisons with the retail meat and human disease was consistent with a role of chickens in human disease, the magnitude of these values probably due to the fact that the *C. jejuni* isolates obtained in this study were not a comprehensive sample of chicken-associated *C. jejuni* in the UK.

This study demonstrates that microbiological, population genetic, behavioural and environmental data can be effectively combined to investigate the transmission and distribution of zoonotic pathogens. None of our findings were consistent with the hypothesis that the free-range environment is a major source of infection of free-range broiler chickens. This conclusion has important implications for the management of *C. jejuni* colonization of chickens in extensive, and perhaps intensive, production systems, suggesting that the impossibility of achieving high biosecurity in extensive production systems may not be the threat to human health that has been feared.

## Experimental procedures

### *Campylobacter jejuni* isolates

In total, 166 *C. jejuni* isolates and one *C. coli* isolate were cultured from faeces from wild geese between August 2002 and February 2003 and 285 *C. jejuni* isolates and six *C. coli* isolates were cultured from faeces from wild starlings (*S. vulgaris*) between July 2002 and February 2005. A total of 419 *C. jejuni* and 448 *C. coli* isolates were cultured from anal swabs from chickens shortly before depopulation at 56 days of age in 2003. Ten birds were sampled from flocks 1–53, and 25 birds were sampled from flocks 54–64. Nucleotide sequence data from 1003 *C. jejuni* and *C. coli* isolates from retail chicken meat in the UK in 2001 and 2005/2006, and 540 *C. jejuni* isolates from human disease in Oxfordshire in 2003/2004 were compared with data from this study.

### Bacterial culture and identification

Faeces from geese and starlings and swabs from chickens were isolated using published enrichment and culture methods (Bolton and Robertson, 1982; Hutchinson and Bolton, 1984; Jorgensen *et al*., 2002). *Campylobacter* colonies were provisionally identified by Gram-negative small curved rod morphology and positive oxidase and catalase reactions and were subcultured onto Columbia horse blood agar (CBA). Species identification was confirmed by ST, resulting from MLST. Chromosomal DNA was extracted from pure cultures grown on CBA using IsoQuick nucleic acid extraction kits, following the manufacturer's instructions for the rapid DNA extraction protocol.

### Multi-locus sequence typing

The original protocol and reaction conditions for MLST were used; however, nucleotide extension reactions were modified to 1/32 size reactions (Dingle *et al*., 2001). The nucleotide extension reaction products were detected on an ABI Prism 3730 automated DNA analyser and assembled using methods described previously (Dingle *et al*., 2001). The consensus sequence was queried against the *Campylobacter* database to give an allele number (Jolley, 2007). The combination of alleles for the seven housekeeping genes gave the ST. Sequence types were assigned into genetically related clusters called clonal complexes, based on sharing four or more alleles with the central genotype that had been identified in previous studies using the burst algorithm and upgma cluster analysis (Feil and Spratt, 2001; Dingle *et al*., 2002).

### Genetic analyses

The pair-wise *F*_ST_and test of significance calculations was performed using arlequin version 3.0. (Wright, 1951; Schneider *et al*., 2000). The sequences at each of the seven loci were concatenated using a tool on the *Campylobacter*MLST database for both of the analyses (Jolley, 2007).

### Chicken production

One-day-old chicks (breed: Sherwood White) were bought in and reared indoors to 24 days, when they were transferred to field arks at one of two commercial free-range farms in Oxfordshire and allowed to range at 28 days. The two farms were about 15 miles apart. At each farm, eight fenced plots (total 16), half with trees and half without, were laid out in a split-plot design, half with 670 chickens in one ark (low range density 1.2 m^2^ per bird) and half with 1340 chickens in two arks (high range density 2.5 m^2^ per bird) (Jones *et al*., 2007). The chickens were grown to a target slaughter weight of 2.5 kg at 56 days and plots were free of chickens for 7 weeks between successive flocks. In total 64 flocks (4 per trial plot) were included in this joint study.

### Chicken health and behaviour variables

The numbers of birds ranging (outside the ark) at 52 days (∼9.30 am); the per cent incidence of poor walking ability (gait), hock burn and pad dermatitis; average flock growth rate and mortality rates of chickens were recorded for each flock (Dawkins *et al*., 2004; Jones *et al*., 2005; 2007).

### Statistical analyses

The outcome variables of per cent carriage rate and diversity of *Campylobacter* were subjected to an analysis of variance General Linear Model (anova, GLM, Minitab version 13.0). The experimental unit was the flock and the experimental model included the fixed effects of site, replicate nested within site, chicken density, and the presence or absence of trees. Response predictor variables (listed above) with significant (*P* < 0.01) correlation to carriage rates and diversity were included stepwise in the model, in order of strength of the Pearson coefficient. Data were tested for normality of residuals and transformed where appropriate. Factors that best explain the observed variation in the outcome variables are given along with the per cent variation explained (*R*^2^). Significant effects of categorical and continuous predictors were further examined by *post hoc* Tukey comparison and regression analysis (fitted line model) respectively. The number of times on which an ST was detected in a flock which ST had also been detected in the previous flock on that plot was counted. The total count was compared with the results of a permutation test. In this test the sets of observed STs were randomly assigned between each of the 16 plots for each of the four cycles of production. The same counting procedure using this randomized data set and the process repeated 10 000 times to estimate the range of counts likely by chance within the data set if no carry-over occurred.
